# A cross-cultural comparison on perception for extended reality (XR) learning environmental simulation of animal drug administration

**DOI:** 10.3389/fvets.2026.1846733

**Published:** 2026-09-03

**Authors:** Eline Wydooghe, Pieter Schutijser, Miu Yung Olivia Ngan, Mau Fung Lee, Chung Hin Kenneth Lai, Chung Lam Wong, Zihan Zhao, Florence Mei Kuen Tang

**Affiliations:** 1 Agro- and Biotechnology, VIVES University of Applied Sciences, Roeselare, Belgium; 2 Educational Technology, VIVES University of Applied Sciences, Brugge, Belgium; 3 Medical Ethics and Humanities Unit, School of Clinical Medicine, LKS Faculty of Medicine, University of Hong Kong, Hong Kong, Hong Kong SAR, China; 4 Information Technology Service Center, The Chinese University of Hong Kong (CUHK), Hong Kong, Hong Kong SAR, China; 5 The Center for eLearning Innovation and Technology, The Chinese University of Hong Kong (CUHK), Hong Kong, Hong Kong SAR, China; 6 The Biomedical Sciences Programme, Faculty of Medicine, The Chinese University of Hong Kong (CUHK), Hong Kong, Hong Kong SAR, China; 7 Division of Biomedical Sciences, School of Biomedical Sciences, The Chinese University of Hong Kong (CUHK), Hong Kong, Hong Kong SAR, China

**Keywords:** 3Rs principle, animal handling, animal-handling training, extended reality, intraperitoneal injection (IP injection), student perception

## Abstract

**Background:**

The integration of Extended Reality (XR) technologies into animal-handling training provides science students working with animals in experimental research with an innovative platform for developing practical and ethical skills that contribute to medical advancement. As laboratory practices become increasingly globalized and guided by the 3Rs principle of Replacement, Reduction, and Refinement, understanding cross-cultural perceptions of animal-handling protocols has become increasingly important.

**Methods:**

A single-player XR platform with an immersive one-in-one virtual reality (VR) system, the Virtual Animal-handling Simulation (*VAnS*), was integrated into aligned curricula at VIVES University of Applied Sciences and the Chinese University of Hong Kong. After lectures and hands-on practice with anatomical dummies, 85 undergraduates completed *VAnS* and then an online questionnaire with 17 questions, comprising six domains: Engagement with 3D World, Realistic Interaction, Active Learning, Concentrated Involvement, Learning Capability, and Overall Perception. Each of the 17 items was rated on a five-point Likert scale.

**Results:**

Median scores in all categories were significantly above the neutral midpoint, indicating strong endorsement of the VR environment, its realism, and its contribution to active learning and perceived capability. Chinese students consistently rated the VR module more favorably than Belgian students across all six learning domains. Effect sizes ranged from small to medium, with the largest differences observed for learning capability and overall perception. Ordinal logistic regression confirmed country as a significant predictor for several domains, while gender showed no meaningful influence. Prior VR experience was negatively associated with Learning Capability (*p* = 0.036). Within the Belgian cohort, animal-care background had no significant effect, whereas educational track showed a non-significant trend toward higher ratings for Learning Capability.

**Conclusion:**

Our study revealed that *VAnS* were widely accepted as an engaging, and 3Rs-aligned supplement to animal-handling training, with students reporting positive perceptions of its potential to support learning. Cultural differences underscoring the need for context-sensitive implementation.

## Introduction

The initials of the 3Rs principle are ‘Replacement', ‘Reduction', and ‘Refinement', provide a framework for performing more humane animal research over 50 years (1–5). Initially proposed as guiding principles to minimize animal use and suffering, the principles have now become a gold standard framework embedded in national and international legislation regulating the use of animals in scientific procedures, subject to approval by ethical committees. In practice, researchers strive to replace mice with non-animal alternatives, such as cell cultures or organoids (6), whenever possible. When mice are necessary, scientists employ sophisticated experimental designs and statistical methods to reduce the number of animals used while still obtaining meaningful results (7). Refinement efforts focus on minimizing discomfort and enhancing mouse welfare through less-invasive techniques, improving housing conditions, and providing pain relief when needed. Advanced technologies, such as imaging tools, allow researchers to track individual mice throughout their lives, reducing overall numbers and refining procedures. Additionally, the development of comprehensive gene activity databases has the potential to prevent the use of thousands of mice in individual experiments by providing readily accessible data. Collectively, the 3Rs principle improves animal welfare and contributes to higher quality, more reproducible science in mouse-based biomedical research (8).

Accordingly, the design of animal experimentation training programmes must integrate not only technical skill development but also ethical reflection, aligning with the principle of the 3Rs (1). In this context, the social value preferences within a society for people's understanding are also crucial for public communication in the acceptance of animal research (9). Across different countries, students approach animal experimentation and welfare through distinct cultural and local legislations, which shape how they perceive animal-handling training. Compared with Europe, many Asian jurisdictions, for example, China, Japan, and Korea, came later to the formal implementation of the 3Rs principle and structured animal-welfare regulation in laboratory animal science, but recent legislative reforms and the establishment of ethics committees indicate a growing openness toward the 3Rs principle and higher welfare standards in animal experimentation (10). Surveys of university students in multiple European and Asian countries show that European cohorts generally express stronger concern for animal welfare and rights and are less accepting of invasive animal use than many Asian cohorts (11). Comparative work on different attitudinal levels of animal welfare in China, Japan, and the Netherlands further indicates that Chinese respondents are most tolerant of using a wide range of species in medical research, whereas Dutch and Japanese respondents display greater welfare awareness and lower overall acceptance of laboratory animal use (12). Still, the 3Rs principle continues to play a pivotal role in the ethical and legal governance of animal experimentation, shaping how students are trained and how they develop attitudes toward animal handling and care. Contemporary legal reviews emphasize that the 3Rs principle is now a central criterion in authorizing the use of animals in research and education, guiding both curriculum design and competency requirements for personnel working with laboratory animals (13, 14).

The digital technology of Extended Reality, encompassing virtual reality (VR), augmented reality (AR), and mixed reality (MR), represents a spectrum of immersive technologies increasingly utilized to transform biomedical sciences education (15–18). VR technology has emerged as a powerful tool for skill training across various fields, including cadet pilot programs, dental surgeon programs (19), and biomedical sciences programs (20–22). The immersive nature of VR enables learners to understand flight zones, practice moving animals safely, and master essential handling techniques without risking injury to themselves or causing stress to real animals.

Recent advancements in VR technology have led to the development of all-in-one headsets that are both user-friendly and capable of supporting multiplayer experiences. For example, the Meta Quest line of headsets offers an immersive, all-in-one VR experience at an affordable price (23). The standalone headsets feature built-in sensors for tracking head and hand movements, allowing users to move freely within a room-scale environment without needing external sensors or cables. The cordless design enhances immersion and reduces the risk of tripping hazards. Many modern VR headsets emphasize social interactions and multiplayer capabilities, allowing users to connect with friends and other players in virtual environments easily (24). The devices often feature smooth, natural motion tracking and easy-to-use interfaces, making them accessible to both casual users and hardcore gamers alike. The ability to engage in multiplayer VR games adds an extra layer of immersion and realism to the experience, with features such as group chats, proximity voice communication, and cross-platform support enabling players to interact seamlessly across different devices.

As animal handling for drug administration is a universal skill, the aim of the study is to collaborate internationally to develop an in-house animal-handling simulation using the latest VR technology. The study objectives are to (a) investigate students perceptions of extended-reality–supported virtual simulation with respect to engagement, active learning, and perceived learning capability within modern biomedical sciences curricula, and (b) explore cross-cultural differences between European and Asian higher-education contexts in students' perceptions of animal experimentation training, the 3Rs principle, and the acceptability of VR as an adjunct or alternative to conventional live-animal experimentations.

## Materials and methods

### XR training simulation game setting

**Figure 1:**
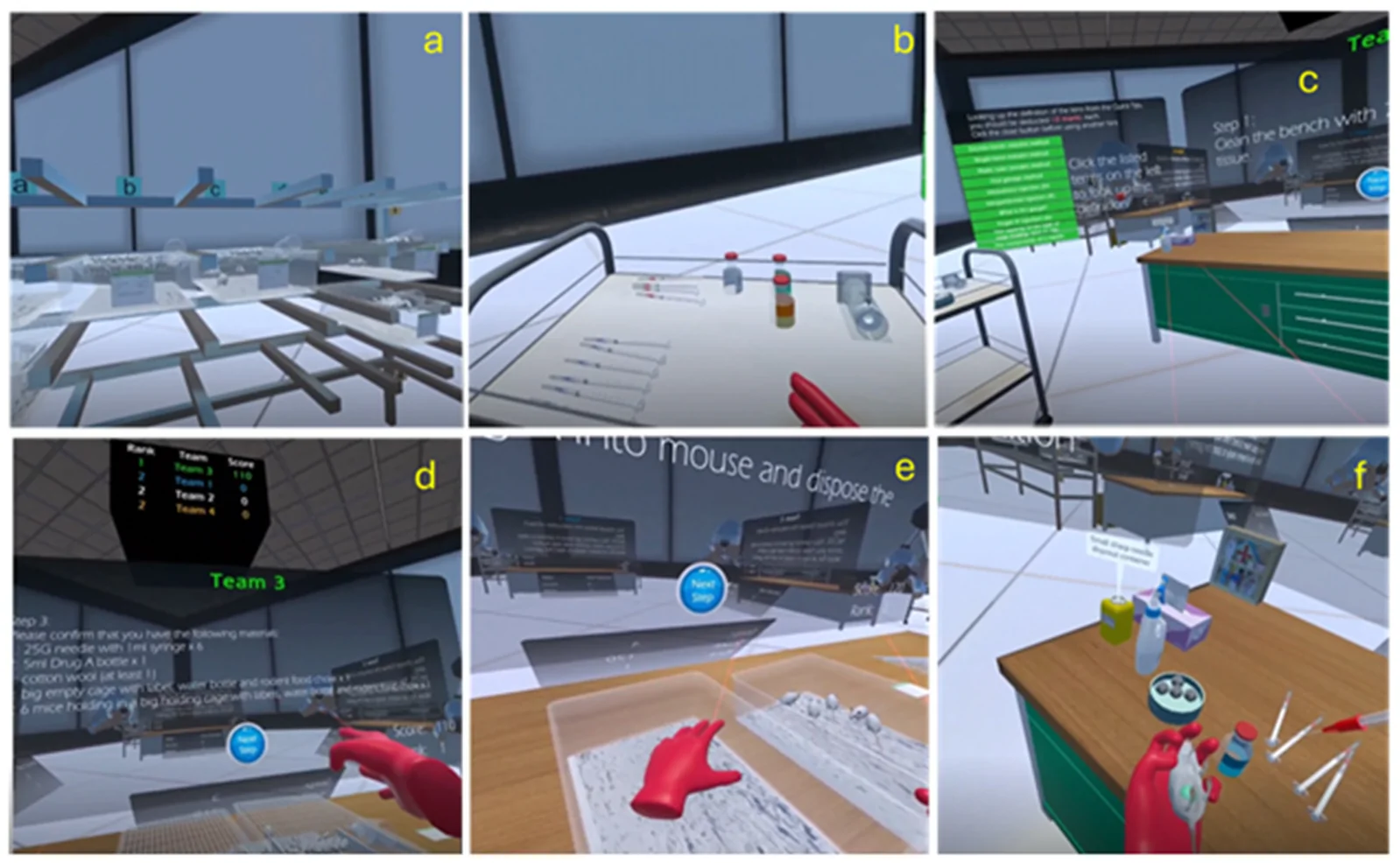
*VAnS* for immersive training in mouse handling and injection procedures aligned with the 3Rs and learner wellbeing. **(a)** Overview of the virtual animal facility where students navigate workstations that illustrate correct cage setup and preparation of the experimental area. **(b)** Close-up of the bench layout showing appropriately labeled syringes, needles of different gauges, and drug vials that students must select before injection. **(c)** On-screen prompts guide learners through bench disinfection, personal protective equipment, and other aseptic preparations before handling animals. **(d)** Single-player game interface and leaderboard in which students follow a stepwise protocol and receive real-time feedback and scoring while preparing for the experiment. **(e)** Simulation of safe mouse handling and transfer between cages so that students can practice animal welfare–oriented techniques without using live animals. **(f)** Virtual practice of drug administration, including dose preparation and needle disposal, reinforcing correct intraperitoneal injection technique and sharps safety.

The Virtual Animal-handling Simulation (*VAnS*) is a virtual reality-based intraperitoneal drug administration game developed at The Chinese University of Hong Kong (CUHK) to provide an immersive, interactive learning experience for healthcare students. It supports training in biomedical research techniques while reinforcing the 3Rs and learner wellbeing by guiding students through (i) correct cage holding and preparation, (ii) appropriate selection of needle gauges for injection, and (iii) stepwise drug administration procedures. Set in a gamified virtual laboratory with a leaderboard, as illustrated in Figure 1, *VAnS* engages learners in friendly competition while they practice essential animal-handling skills in a risk-free environment.

### Implementation of the *VAnS* gamification at the two universities

In the academic year 2024–25, *VAnS* was implemented at both universities. Participants were students enrolled in the (i) Bachelor of Agro- and Biotechnology programme at VIVES University of Applied Sciences, specializing in Animal Care (on-campus or distance learning) or Biotechnology, and (ii) Bachelor of Biomedical Sciences Programme (BMS) at CUHK of the Faculty of Medicine, specializing in animal research studies in medical treatments. The integration of *VAnS* comprised three sequential phases: (i) theoretical lectures, (ii) hands-on training with anatomical dummies, and (iii) XR simulations of mouse handling and intraperitoneal (IP) injections. Within the XR module, students entered a virtual laboratory and completed a realistic sequence of tasks, including disinfecting the workspace, selecting the required materials, and preparing the appropriate cage of mice. With the *VAnS* immersive training, they then individually handled the animals, administered an IP injection of 0.25 ml of Drug A to six mice, and transferred each mouse to an empty cage after the procedure.

### Evaluation of *VAnS* study

An online survey was designed to evaluate a single-player XR platform developed at the Chinese University of Hong Kong and implemented in the curriculum. The survey first collected demographic and background information of the student, including country, gender and educational track. In the second part, a 17-item scale comprised of six domains assessing student perceptions of the XR application (Table 1): Engagement with 3D World (E3DW), Realistic Interaction (RI), Active Learning (AL), Concentrated Involvement (CI), Learning Capability (LC), and Overall Perception (OP). Each statement was rated on a five-point Likert scale ranging from strongly disagree to strongly agree. A composite score was calculated by summing the responses in the respective domain.

**Table 1:** The content of the online eSurvey questionnaire used to evaluate students' perceptions of the *VAnS* virtual animal-handling simulation.

Engagement with 3D world (E3DW)	
1.	The immersive VR creates a realistic-looking lab environment.
2.	The immersive VR system gives me a perceptual feeling to manipulate the 3D objects.
3.	I can psychologically study in the lab when using the immersive VR system.
Realistic interaction (RI)	
4.	I can recall myself in the interaction with the virtual mice by playing the immersive VR system
5.	The VR system can enhance my knowledge of the virtual learning environment.
Active learning (AL)	
6.	The VR system helps me understand the learning content beyond the lecture.
7.	The VR system can motivate me to learn more.
8.	I like to have a gamified learning arrangement in the class with competition.
Concentrated involvement (CI)	
9.	The VR system can deepen my knowledge acquisition.
10.	The VR system can make me enjoy and embrace the learning process.
Learning capability (LC)	
11.	The VR system can facilitate my learning capability.
12.	The immersive VR system gives me the perceptual feeling of manipulating the 3D objects.
13.	The VR system can consolidate my memory in knowledge.
14.	The VR system can migrate my theoretical knowledge to practical application.
15.	It can provide other opportunities to work with technology for interactive learning.
Overall perception (OP)	
16.	I believe that the learning space added value to consolidate the learning progress.
17.	Overall, VR gamification can be my learning preference.

### Statistical analyses

All analyses were conducted using IBM SPSS Statistics (version 29). Statistical significance was set at *p* < 0.05. Cross-cultural differences in student perceptions of the XR application were initially examined using Mann–Whitney U tests. Effect sizes for these comparisons were calculated using the *r* statistic and Cliff's δ Effect sizes are also reported alongside *p*-values for all primary comparisons to characterize the magnitude of group differences and to guard against spurious Type I error inferences based on *p*-values alone (25, 26). These measures provide insight into practical significance beyond *p*-values: *r* expresses the standardized strength of association, while δ reflects the probability that a randomly selected student from one group scores higher than a student from the other group. Effect-size magnitudes were interpreted using established thresholds: *r* ≈ 0.10 (small), 0.30 (medium), and 0.50 (large); Cliff's δ ≈ 0.147 (small), 0.33 (medium), and 0.474 (large) (27). For ordinal logistic regression models, odds ratios (ORs) with 95% confidence intervals are reported as the effect-size measure, with |OR| ≈ 1.5/2.5/4.5 corresponding to small/medium/large effects (28). To facilitate ordinal regression analysis and reduce sparse cell frequencies, the composite scores of each domain were subsequently grouped into three ordered categories (low, medium, high) based on the 33rd and 66th percentiles of the distribution within the full sample. Separate ordinal regression models (logit link) were then fitted for each domain, with the domain score as the dependent variable, Country as the main predictor, and Gender and Prior VR experience as covariates. Results are reported as odds ratios (OR) with 95% confidence intervals. The proportional odds assumption was evaluated using the Test of Parallel Lines. In addition, for the Belgian cohort only, where variation existed in educational track (distance learning vs. regular track) and animal care and handling experience, exploratory analyses were conducted to assess whether these factors were associated with domain scores. These additional analyses were not performed for the Chinese cohort, as all students belonged to the same program and reported similar VR experiences.

## Results

### Study population

A total of *N* = 85 students participated in the study, including *n* = 43 Chinese students from BMS, CUHK, SAR, and *n* = 42 Belgian students from VIVES University. Demographic and educational characteristics were collected for all participants, including gender, educational track (distance learning vs. regular track), animal care and handling experience, and prior VR experience (Table 2).

**Table 2:** Demographics of the participating students.

Demographics		China (CUHK) *n* (%)	Belgium (VIVES) *n* (%)	Overall *n* (%)
Gender	Male	20 (46.5%)	11 (26.2%)	31 (36.5%)
	Female	23 (53.5%)	29 (69.1%)	52 (61.2%)
	Transgender	0 (0%)	2 (4.7%)	2 (2.3%)
Educational track	Regular Track (In-person)	43 (100%)	27 (64.3%)	70 (82.4%)
	Distance Learning	0 (0%)	15 (35.7%)	15 (17.6%)
Animal care experience	Yes	0 (0%)	6 (14.3%)	6 (7.1%)
	No	43 (100%)	36 (85.7%)	79 (92.9%)
Prior VR-experience	Yes	35 (81.4%)	26 (61.9%)	61 (71.8%)
	No	8 (18.6%)	16 (38.1%)	24 (28.2%)
TOTAL		43	42	85

### Cross-cultural differences in student perceptions

Differences between Chinese and Belgian students were examined across the six domains of the online questionnaire (*N* = 85). For each domain, a composite score was calculated by summing the Likert-scale responses to all items in that domain. Chinese students (*n* = 43) consistently demonstrated higher mean ranks than Belgian students (*n* = 42), indicating more favorable perceptions of the VR module (Table 3). For example, in Engagement with the 3D world, the mean rank for Chinese participants was higher compared to Belgian participants (48.23 vs. 37.64; *p* = 0.046; *r* = 0.23; δ ≈ 0.21, small effect). Similar patterns were observed for Realistic Interaction 48.48 vs. 37.39; *p* = 0.032; *r* = 0.23; δ ≈ 0.21, small effect), Active Learning (41.85 vs. 32.09; *p* = 0.050; *r* = 0.22; δ ≈ 0.20, small effect; total *n* = 74) and Concentrated Involvement (48.17 vs. 37.70; *p* = 0.044; *r* = 0.23; δ ≈ 0.21, small effect). Notably, Learning Capability (52.31 vs. 33.46; *p* < 0.001; *r* = 0.38; δ = 0.44, medium effect) and Overall Perception (44.39 vs. 28.94; *p* = 0.002; *r* = 0.36; δ = 0.42, medium effect) showed medium-sized effects.

**Table 3:** Cross-cultural comparison of student perceptions of a VR application across the six domains.

Outcome	Country	*N*	Mean rank	*P* (2-tailed)	*R* (^**^)	Cliff's δ (^***^)
Engagement with 3D world	China	43	48.23	0.046	0.22 (small)	0.25 (small)
	Belgium	42	37.64			
Realistic interaction	China	43	48.48	0.032	0.23 (small)	0.26 (small)
	Belgium	42	37.39			
Active learning^*^	China	41	41.85	0.050	0.23 (small)	0.26 (small)
	Belgium	33	32.09			
Concentrated involvement	China	43	48.17	0.044	0.22 (small)	0.25 (small)
	Belgium	42	37.70			
Learning capability	China	43	52.31	< 0.001	0.38 (medium)	0.44 (medium)
	Belgium	42	33.46			
Sum overall perception^*^	China	41	44.39	0.002	0.36 (medium)	0.42 (medium)
	Belgium	33	28.94			

^*^Available-case analysis (total *N* = 74).

^**^Thresholds: 0.10 small, 0.30 medium, 0.50 large.

^***^Cliff's δ thresholds: 0.147 small, 0.33 medium, 0.474 large.

### Adjusted ordinal logistic regression analyses across VR domains

To account for potential confounding factors, ordinal logistic regression models were fitted for each domain, with Country as the main predictor and Gender and Prior VR Experience as covariates (*N* = 85). Composite scores were categorized into three ordered levels (low, medium, and high) based on the 33rd and 66th percentiles of the full sample distribution. The proportional odds assumption was met for all models (Test of Parallel Lines, *p* > 0.05).

Country was a significant predictor for Learning Capability (OR = 5.17, 95% CI: 2.00–13.36, *p* < 0.001, large effect), Realistic Interaction (OR = 3.03, 95% CI: 1.18–7.81, *p* = 0.021, medium effect), and Overall Perception (OR = 2.84, 95% CI: 1.10–7.34, *p* = 0.031, medium effect). Trend-level associations were observed for Engagement with 3D World and Active Learning, while Concentrated Involvement (CI) did not reach statistical significance (Table 4).

**Table 4:** Effects of Country, Gender and Prior VR-Experience on the outcome of each of the six domains assessing student perception.

Outcome	Predictor	Odds ratio (OR)	95% CI (OR)	*p*-value	Effect size (OR magnitude)
Engagement with 3D World	Country	2.27	[0.92; 5.58]	0.074^**^	small
	Gender	0.82	[0.36; 1.88]	0.644	negligible
	Prior VR-experience	0.64	[0.25; 1.67]	0.364	small
Realistic interaction	Country	3.03	[1.18; 7.81]	0.021^*^	medium
	Gender	0.59	[0.25; 1.40]	0.234	small
	Prior VR-experience	0.55	[0.21; 1.46]	0.230	small
Active learning	Country	2.47	[0.93; 6.60]	0.071^†^	small
	Gender	0.55	[0.22; 1.33]	0.185	small
	Prior VR-experience	0.58	[0.20; 1.63]	0.300	small
Concentrated involvement	Country	2.05	[0.84; 5.00]	0.115	small
	Gender	0.70	[0.31; 1.60]	0.395	negligible
	Prior VR-experience	0.56	[0.21; 1.44]	0.228	small
Learning capability	Country	5.17	[2.00; 13.36]	< 0.001^*^	large
	Gender	0.60	[0.26; 1.37]	0.221	small
	Prior VR-experience	0.35	[0.13; 0.94]	0.036^*^	medium
Overall perception	Country	2.84	[1.10; 7.34]	0.031^*^	medium
	Gender	0.68	[0.29; 1.60]	0.373	negligible
	Prior VR-experience	0.76	[0.28; 2.09]	0.601	negligible

Reference categories: Country = Belgium; Gender = male; Prior VR-Experience = 0 (no prior VR experience). Odds ratios and 95% CIs were obtained by exponentiating parameter estimates and confidence limits from SPSS.

^*^p < 0.05 (statistically significant).

^†^ 0.05 ≤ *p* < 0.10 (trend-level association).

Gender was not significantly associated with any domain (all *p* > 0.18). While prior VR experience was negatively associated with Learning Capability (OR = 0.35, 95% CI = 0.13–0.94, *p* = 0.036, medium effect), indicating that students without prior VR experience had higher odds of reporting higher learning capability scores. This association represents a medium effect size based on established thresholds.

### Effects of educational track and animal care experience on VR perception of the Belgian students

Because there was no variability in educational track or animal care experience among the Chinese students, these analyses could only be conducted within the Belgian subsample (*n* = 42). Effects of educational track and animal care experience on VR perception of the Belgian students were examined using ordinal regression. The analyses revealed no statistically significant effects of animal care background or educational track on students' perceptions of the VR application across all six domains [all *p* >.05 (Table 5)]. However, in the domain *Learning Capability*, students in the regular track had an OR of 3.99 (95% CI = 0.84–18.91, *p* = 0.082, medium effect), indicating higher odds of reporting higher scores among students in the regular track, although this estimate was imprecise and not statistically significant. Similarly, animal care background showed no meaningful effect (ORs between 0.63 and 2.21, *p* > 0.05, negligible to small effect). These findings suggest no clear evidence that prior experience and program type substantially influenced VR perception among Belgian students.

**Table 5:** Ordinal regression results for the effects of animal care experience and educational track on six domains of VR perception among Belgian students.

Domain	Predictor	Odds Ratio (OR)	95% CI (OR)	*p*-value	Effect Size (OR magnitude)
Engagement with 3D World	Animal care experience	1.54	[0.20; 11.67]	0.678	Small
	Educational track	1.97	[0.44; 8.79]	0.375	Small
Realistic interaction	Animal care experience	1.21	[0.16; 9.12]	0.854	Negligible
	Educational track	2.98	[0.66; 13.49]	0.156	Medium
Active learning	Animal care experience	2.21	[0.22; 30.07]	0.502	Small
	Educational track	3.16	[0.63; 15.66]	0.160	Medium
Concentrated involvement	Animal care experience	0.80	[0.11; 6.00]	0.826	Negligible
	Educational track	2.38	[0.56; 10.19]	0.242	Small
Learning capability	Animal care experience	0.63	[0.08; 4.99]	0.663	Small
	Educational track	3.99	[0.84; 18.91]	0.082^†^	Medium
Overall perception	Animal care experience	1.71	[0.18; 16.57]	0.642	Small
	Educational track	1.20	[0.27; 5.19]	0.809	Negligible

Odds ratios and 95% CIs were obtained by exponentiating parameter estimates and confidence limits from SPSS.

^†^0.05 ≤ *p* < 0.10 (trend-level association).

## Discussion and conclusion

Notably, XR technology has emerged as a transformative tool in skill training, providing immersive, interactive experiences that overcome traditional barriers to teaching. This study demonstrates that students perceive immersive gamified simulation, using *VAnS* as an example, as a useful tool for ethical and effective skills training in laboratory animal science. By enabling students to practice complex procedures, such as intraperitoneal injection, in a risk-free, animal-free environment, *VAnS* addresses a critical gap in early-stage training. More importantly, in this pilot quantitative study, students reported high levels in all six domains (Engagement with 3D World, Realistic Interaction, Active Learning, Concentrated Involvement, Learning Capability, and Overall Perception) in the context of the cross-cultural skill training, reinforcing the perceived pedagogical value of immersive technologies. These self-reported perceptions are consistent with previous studies suggesting that XR-based education can enhance procedural competence, confidence, and knowledge retention (7, 29, 30); however, the present study did not include objective measures to evaluate such outcomes. Importantly, these findings support the operationalization of the 3Rs principle: by minimizing reliance on live animals, lowering the number required for proficiency, and enhancing welfare through virtual pre-training (31–33). Substituting digital animals for early-stage training is supported by literature reviews and papers showing that XR-driven virtual animal-handling training for drug-injection training provide a risk-free environment where students can practice repeatedly, strengthening confidence and procedural competence before working with live animals (21, 34, 35).

All measured domains received high Likert-scale ratings, indicating strong overall acceptance of the VR module. The results of this study demonstrate strong Engagement with 3D World (mean = 10.34/15) and high ratings for Realistic Interaction (mean = 7.31/10). These findings indicate that the virtual laboratory and animal models successfully created a sense of “being there,” consistent with studies reporting that immersive VR promotes psychological presence and deeper cognitive processing in scientific simulations (36). Prior work has also shown that interactive 3D models enhance spatial reasoning and long-term retention, supporting the interpretation that realistic virtual handling fosters durable procedural memory.

The cross-cultural comparison between Chinese and Belgian students revealed systematic differences in students' perceptions of the same VR module. Chinese students consistently reported higher scores for all investigated domains, suggesting that, besides contextual factors such as local curriculum integration and institutional technology readiness, culturally shaped attitudes toward animal use and welfare may also influence the uptake and perceived value of XR training. These patterns are consistent with broader surveys showing that European students often express stronger ethical reservations about animal use, whereas Asian cohorts may be more accepting of animal experimentation but respond very positively to VR once its educational and welfare benefits are emphasized (11). These findings suggest that while XR-driven animal-handling simulations are well-received and perceived as valuable by students, their design and implementation should account for cultural differences in educational contexts and animal welfare perspectives to maximize impact across diverse learning environments.

Regression analysis provides important insights into the factors influencing students' perceptions of *VAnS* training, including personal characteristics as gender and prior VR experience. When controlling for these characteristics, country remained a significant predictor across several domains, including Learning Capability, Realistic Interaction, and Overall Perception, with odds ratios indicating that Chinese students were substantially more likely to report high scores than the Belgian students in this study. Interestingly, prior VR experience was negatively associated with Learning Capability, suggesting that students without prior VR experience perceived greater learning benefits. This may reflect a novelty effect or heightened engagement among first-time users, a phenomenon reported in other XR studies. For example, Miguel-Alonso et al. (37) documented that initial exposure to VR significantly increased satisfaction, engagement, and immersion, attributing this to the novelty effect, which can influence early learning experiences. Similarly, Huang (38) observed that motivation and engagement remain high during initial sessions despite a gradual decline in novelty, emphasizing that thoughtful instructional design can sustain these benefits over time. These findings underscore the importance of considering user familiarity when evaluating VR's impact and designing curricula that balance novelty-based in interactive engagement with long-term learning objectives. Gender did not significantly influence any domain in this study, suggesting that this XR-driven training offers an equitable learning experience across male and female students. This contrasts with earlier XR and simulation-based training studies, such as Ausburn et al. (39), who found that in desktop VR environments, female learners often reported lower confidence and comfort than male learners, even when objective performance metrics were similar, suggesting that gendered expectations and prior exposure to digital games may shape self-reported learning gains more than the actual usability of the system. These results underline the importance of designing XR-driven animal-handling training that explicitly supports all genders, for example, by scaffolding early tasks, offering clear performance feedback, and fostering inclusive group dynamics, to ensure that perceived learning capability aligns with actual competence across the whole cohort.

Within the Belgian cohort, ordinal regression analyses revealed no statistically significant effects of animal care experience or educational track on students' perceptions of the XR-driven training across the six investigated domains. Only a trend could be observed for regular-track students to rate Learning Capability higher. This aligns with previous research indicating that VR can act as an equalizer by reducing disparities linked to experiential background, particularly in early-stage practical training (21, 40–42). From the data analysis, all students, irrespective of prior animal-care experience, received theoretical instruction on the specific procedure and practiced the technique on physical dummies before engaging with the VR module. This structured progression likely contributed to the consistency of perceptions across backgrounds, supporting the interpretation that VR complements, rather than replaces, foundational learning steps. Although such outcomes were not directly assessed in the present study, prior research suggests that XR-based training may enhance declarative knowledge and support skill transfer to real-world settings. For instance, Yu et al. (43) demonstrated that high-immersion VR significantly enhances both declarative and procedural knowledge compared to less immersive formats, while Cho and Kim's (44–47) meta-analysis in nursing education reported large effect sizes for VR-based improvements in competency and clinical performance. For curriculum design, this implies that XR-driven training can be effectively integrated across diverse student populations without requiring major adaptations based on prior experience, thereby promoting inclusivity and standardization in laboratory animal science education.

### Limitations and future directions

Several limitations should be acknowledged in interpreting these findings. First, this study was not designed to evaluate educational effectiveness, as it included no objective performance measures (e.g., procedural accuracy, task performance, knowledge retention, or transfer to practice) or comparison groups. In addition, the absence of a control group precludes isolating the added educational value of the VR intervention compared to traditional or alternative instructional methods. Furthermore, the study used a cross-sectional, single-cohort design and therefore cannot determine long-term skill retention or the extent to which competence transfers into real-world practice; prior work on simulation-based and immersive XR-driven training interventions similarly highlights the need for more rigorous, longitudinal, and multi-center research that triangulates perception data with objective performance outcomes (48, 49). Second, all outcomes were learners‘ perceptions reported on Likert-type scales, which are vulnerable to response biases such as social desirability and acquiescence, and the modest sample size (*N* = 85) limits the precision of subgroup estimates. Third, although the XR-driven training intervention, measurement instrument, and analytic framework were intentionally standardized across the two institutions, differences in local curriculum-making processes, stakeholder configurations, and broader system-level conditions are known to shape educational implementation and outcomes, constraining direct cross-institutional comparability (50). At the faculty level, variation in educators' attitudes toward and engagement with educational research and innovation can further influence how such initiatives are framed, supported, and taken up within programmes (51). Fourth, consumer-grade head-mounted VR displays still impose persistent technical constraints, i.e., limited haptic realism, residual motion sickness, and reduced fidelity of small-animal anatomical models, which may attenuate transfer to real-world procedural settings. Future work should therefore prioritize longitudinal randomized controlled trials with pre-registered methodologies and standardized outcome measures (52), coupled with advanced haptic integration, adaptive feedback, and culturally responsive design strategies to ensure inclusivity across modest sample sizes in institutions worldwide.

A specific design limitation of the present study is that *VAnS* was implemented as a gamified virtual laboratory with a leaderboard, scoring, and stepwise task prompts, rather than as a first-person point-of-view (*FP-POV*) immersive recording. *FP-POV* approaches use stereoscopic 180° video captured from the operator's own perspective and are hypothesized to support motor-skill acquisition through mirror-neuron-mediated observational learning (53, 54). Empirical comparisons have shown that *FP-POV* can outperform third-person video in immersive VR environment with the non-immersive video or screen-based formats have been used to support training in stereotaxic surgery (53), subdermal drain placement on cadavers (55), and communication in distributed emergency medical teams (56). By contrast, the engagement and knowledge benefits of XR-driven gamified VR, which has been well documented in meta-analyses (57) and in systematic reviews of game-based learning theory (58), may not generalize as directly to embodied, sensorimotor animal-handling skill, consistent with predictions of the cognitive affective model of immersive learning (59). *VAnS* is therefore best understood as a gamified preparatory tool that complements, rather than substitutes for, *FP-POV* immersive simulations and live-animal supervised practice. Future iterations of *VAnS* will incorporate *FP-POV* stereoscopic recordings of expert handlers performing intraperitoneal injections and compare gamified-only with live-handling control groups, presented by emerging evidence on *FP-POV* in anesthesia education and simulation.

Beyond the contrast between *FP-POV* and gamified design, two further considerations shape the next generation of XR-driven gamified simulation. First, the integration of adaptive-AI feature into XR-driven training enables personalized feedback and learner profiling. However, it also raises ethical and regulatory concerns, including algorithmic bias, reduced learner autonomy, biometric data privacy, and overreliance on AI scaffolding. These challenges must be addressed through transparent opt-in design and human-in-the-loop oversight (60). Cross-cultural evidence further indicates that learners from different educational and linguistic backgrounds adopt and interpret generative AI tools differently, underscoring the need for culturally calibrated AI scaffolds in any future adaptive-AI XR-driven training deployment (61). Second, the 10-min module length is a deliberate instructional-design choice, grounded in evidence that focused attention during didactic delivery declines after approximately 10 min (62, 63). Accordingly, XR-driven training modules should be chunked into around 10-min task segments, interleaved with reflective pauses and debriefing, regardless of whether the underlying platform is *FP-POV* observational, gamified or adaptive AI-augmented.

Overall, the study reflects a coherent pedagogical strategy for integrating XR technologies into animal-handling training. The findings indicate that students perceive XR-based applications as promoting interactive learning and supporting cognitive engagement. Future XR-drive training developments should consider enhancements such as pre-training modules to familiarize cross-regional learners with key terminology and controls, integration of *FP-POV* multimedia to support knowledge acquisition, AI-augmented guidance to address misconceptions, and segmentation of content into short, learner-paced chunks (64).

Regarding the 3Rs principle, the application of *VAnS* suggests that XR-driven training in mouse handling and injection procedures is a promising and widely accepted approach in veterinary and biomedical education, offering benefits such as enhanced safety, ethical compliance, and virtual hands-on experience. By enabling students to practice complex procedures in a controlled environment, these tools complement rather than replace traditional hands-on training, supporting a balanced curriculum that supports skill mastery and animal welfare standards. As further XR technology evolves, improvements of hardware in haptic realism, integration of *FP-POV* and AI-augmented elements will further strengthen XR-driven the gamified platform. Future research should prioritize longitudinal study designs, objective performance assessments, and inclusive approaches to evaluate learning outcomes and ensure equitable educational impact. Expanding access to advanced information and communication technologies may further support the integration of XR-driven training as a component of modern biomedical education, while upholding the 3Rs principle and promoting high standards for ethical laboratory training worldwide.

## Data Availability

The original contributions presented in the study are included in the article/supplementary material, further inquiries can be directed to the corresponding author.
